# PRESERVING THE DISCRETENESS OF DEFICITS LEADS TO LOWER FRAILTY INDEX IN INDIVIDUALS LIVING IN LONG-TERM CARE

**DOI:** 10.1093/geroni/igad104.2296

**Published:** 2023-12-21

**Authors:** Brian Greeley, Hilary Low, Ronald Kelly, Robert McDermid, Xiaowei Song

**Affiliations:** Fraser Health, Surrey, British Columbia, Canada; Fraser Health, Surrey, British Columbia, Canada; Fraser Health Authority, Surrey, British Columbia, Canada; Fraser Health, Surrey, British Columbia, Canada; Fraser Health, Surrey, British Columbia, Canada

## Abstract

The frailty index (FI), based on the deficit accumulation model, has potential to advance healthcare, but conventional coding of raw scores introduces noise. This study assesses the impact of the two different coding approaches on the FI. Two FI were calculated using 43 variables from 29,758 older (> 65 years old) Canadians adults (84.6 ± 8 years old; 64% female) living in long-term care. Scores were coded as 0, 0.5, or 1 regardless of the number of levels (grouped), or preserved (e.g., a 4 level variable was coded as 0, 0.33, 0.67, or 1; discrete). FI was correlated to age. Each ordinal variable was removed from the FI to further test the impact of the two coding approaches. The median FI for the grouped approach (0.302 (0.221 – 0.372)) was higher relative to the discrete approach (0.237 (0.170 - 0.307)). The discrete (r = .91) and grouped (r = .93) FI showed similar relationships to age. Removal of any ordinal variable reduced the grouped FI it by 0.004 or 0.016, whereas removal lead to both increases (range: 0.003 - 0.001) and reductions (range: 0.002 - 0.008) for the discrete FI. Using a grouped coding approach when quantifying frailty status artificially inflates FI among a large sample of older Canadians adults living in long-term care. The study underscores the importance of future FI research to adopt a discrete coding approach that accurately reflects the true level of impairment, for reducing noise in statistical models and better clinical utility.

